# Mesonephric adenocarcinomas in female genital tract: A case series

**DOI:** 10.1097/MD.0000000000027174

**Published:** 2021-09-03

**Authors:** Chuan Xie, Qiuhe Chen, Yangmei Shen

**Affiliations:** a Department of Gynecology and Obstetrics, West China Second University Hospital, Sichuan University, China; b Key Laboratory of Birth Defects and Related Diseases of Women and Children, Ministry of Education, Sichuan University, China; c Department of Pathology, West China Second University Hospital, Sichuan University, China.

**Keywords:** case series, mesonephric adenocarcinoma, mesonephric-like adenocarcinoma, treatment

## Abstract

Mesonephric adenocarcinoma (MNAC) is a very rare tumor that originates from mesonephric duct remnants of the female genital tract. Only a few cases were reported in the literature, and most of them occurred in the cervix, extremely rare in the uterine body and ovary. MNAC was rarely reported to arise in the uterine corpus, but never was reported in the ovary. Mesonephric-like adenocarcinomas are recently suggested to describe these neoplasms arising from the uterine corpus and ovary. Due to the rareness of the disease, little is known regarding clinical characteristics, pathological diagnosis, prognosis, and optimal management strategy of MNAC in the female reproductive system. We report a series of MNACs arising from the vagina, cervix, uterine corpus, ovary, and fallopian tube, to summarize the clinical characteristics, pathological diagnosis, treatment, and prognosis.

We retrospectively analyzed all MNACs in the female genital tract derived from our institute from January 2010 till January 2020. Patients’ clinical details and follow-up were obtained from hospital records and scans were obtained from picture archiving and communication system.

A total of 11 patients were included. The median age of onset of symptoms was 52 years. All patients underwent total hysterectomy and bilateral salpingo-oophorectomy, and lymph node dissections were performed in 7/11 (63.6%) patients. Two/eleven (18.2%) received neoadjuvant chemotherapy before surgery and 7/11 (63.6%) received adjuvant chemotherapy after primary surgery. Of the 11 patients, only 1 patient received adjuvant radiation therapy. One patient died at the end point of this study, 9 patients (81.8%) survived and 1 patient was lost to follow-up. The mean follow-up duration was 33.5 months.

Although there is no consensus for the optimal treatment of this rare disease, radical surgery is considered to be the initial choice for localized lesion. Given the high malignancy, the majority of MNAC or mesonephric-like adenocarcinoma patients who underwent adjuvant chemotherapy received 4 to 8 cycles of carboplatin/paclitaxel as a first-line treatment after primary surgery with a median progression-free survival of 12 months. Treatment for recurrent disease in these patients included gemcitabine, carboplatin, and paclitaxel. Radiation was very limited in the treatment of the disease.

## 1 Introduction

Mesonephric adenocarcinoma (MNAC) is an extremely rare and highly malignant tumor in the female reproductive system. It is considered to originate from the embryonal remnants of mesonephric ducts (also known as Wolffian ducts).^[1,2]^ During embryonic development, the females have 2 sets of paired primitive reproductive ducts: the para-mesonephric (Müllerian) and the mesonephric (Wolffian) ducts. In the embryologic females, Müllerian ducts become the female reproductive ducts, while the mesonephric ducts degenerate.^[3]^ However, vestiges of mesonephric ducts may persist along the female genital tract in the form of epithelial inclusions which are called mesonephric remnants.^[4]^ The embryological remnants are found pre-dominantly in the para-ovarian region (epoophoron and paroophoron) and deep in the cervical stroma in the lateral walls. Therefore, MNACs in the female genital tract occur most commonly in the cervix and vagina, and less frequently in the upper female genital tract.^[5]^ MNACs was rarely reported to arise in the uterine corpus, but was never reported in the ovary. It was reported that adenocarcinomas of endometrium and ovary shared morphologic, immuonphenotypic, and molecular features with MNAC, but were lack of association with mesonepheric remnants or hyperplasia.^[6]^ Hence, mesonephric-like adenocarcinomas (MLAs) are recently defined as tumors exhibiting the classic morphologic features of mesonephric carcinoma, but occurring outside of the cervix and without convincing mesonephric remnants.^[4,7–9]^

Due to the paucity of available data, little is known regarding the clinical characteristics, pathological diagnosis, prognosis, and optimal management strategy of MNAC and MLA in the female reproductive system. To add to existing literature, we performed a retrospective review of 11 cases of MNAC and MLA diagnosed and treated at a single institution between January 2010 and January 2020. The purpose of this case series is to summarize the clinical characteristics, pathological diagnosis, treatment, and prognosis of MNAC and MLA in the female genital tract.

## 2 Materials and methods

All MNACs and MLAs were retrieved from the Anatomical Pathology Department at West China Second University Hospital, Sichuan University. We retrospectively analyzed all MNACs in the female reproductive system diagnosed at our institute from January 2010 to January 2020 (The disease is nearly undiagnosed before 2010 in our hospital because of a lack of awareness of the disease). Patients’ clinical characteristics and follow-up were obtained from hospital records and scans were obtained from picture archiving and communication system. The age at diagnosis, presenting symptom, surgical approach, FIGO stage, chemotherapy regimen, histopathology, and follow-up of all the patients were analyzed. All surgical specimens were re-evaluated by 2 specialized gynecologic pathologists. According to the diagnostic criteria for MNAC, patients whose surgical specimens showed characteristic morphologic features of MNAC as well as either immunohistochemical confirmation or molecular confirmation, were included in this study. Patients with the following conditions were excluded: 1 or 2 specialized gynecologic pathologists deny the diagnosis, patients without survival data or complete follow-up data.

Progression-free survival (PFS) was defined as the time interval from initial surgery to the first radiologic or biopsy-proven disease recurrence, or the last follow-up visit in the absence of recurrent disease. Overall survival was measured as the time interval from initial surgery to death.

All experimental protocols were approved by the ethics committee of West China Second University Hospital, and the methods were carried out in accordance with the relevant guidelines and regulations. Informed consent was obtained from all participants.

## 3 Results

A total of 15 patients were retrieved. Among them, 1 was excluded after being confirmed by 2 pathologists, 3 patients without the survival data were excluded. Eleven patients who met the inclusion criteria were ultimately included.

### 3.1 Clinicopathologic characteristics of MNACs in the female genital tract

Among the 11 patients, there were 2 ovarian MLAs, 5 MLAs of uterine corpus, 1 primary oviducal MNAC, 2 cervical MNACs, and 1 vaginal MNAC. Clinical features are summarized in Table 1.

**Table 1:** Clinicopathologic features of mesonephric adenocarcinoma in female genital tract.

Case number	Age	Site	Presenting symptom	CA125^∗^ (U/mL)	FIGO stage	Surgery	Adjuvant chemotherapy (# cycles); recurrence treatment (# cycles)	Clinical outcomes
1	45	Fallopian tube	Left lower abdominal pain	16.6	IA	HYS + BSO + LND + omentectomy	PC (4 cycles); GC (5 cycles)	PFS2^†^: 22 months; no evidence of disease
2	29	Ovary	Abdominal discomfort	13	IC	HYS + BSO + LND + omentectomy	PC (6 cycles)	PFS: 13 months; no evidence of disease
3	56	Ovary	Abdominal discomfort	25.3	IC	HYS + BSO + LND + omentectomy	PC (6 cycles)	PFS: 8 months; no evidence of disease
4	75	Uterine corpus	Vaginal bleeding	8.1	IIIA	HYS + BSO	DC (NACT: 2 cycles, PACT: 6 cycles)	PFS: 10 months; no evidence of disease
5	55	Uterine corpus	Vaginal bleeding	145.1	IVB	HYS + BSO	IC (NACT: 2 cycles, PACT: 6 cycles); RT	PFS: 11 months, OS: 12 months; died of disease
6	67	Uterine corpus	Vaginal bleeding	20.5	IB	HYS + BSO + LND	PC (5 cycles)	PFS: 8 months; no evidence of disease
7	54	Uterine corpus	Vaginal bleeding	7.2	IIIC	HYS + BSO + LND	PC (6 cycles)	PFS: 12 months; no evidence of disease
8	60	Uterine corpus	Vaginal discharge	NA	IB	HYS + BSO	N	PFS: 61 months; alive with disease; lost to follow-up
9	31	Vagina	Vaginal discomfort	11.5	IB	HYS + BSO	PC (8 cycles)	PFS: 90 months; no evidence of disease
10	50	Cervix	Vaginal bleeding	NA	IB1	Radical HYS + BSO	N	PFS: 64 months; no evidence of disease
11	49	Cervix	Vaginal bleeding	37.3	IB1	Radical HYS + BSO + LND	N	PFS: 70 months; no evidence of disease

BSO = bilateral salpingo-oophorectomy, DC = docetaxel and carboplatin, GC = gemcitabine and carboplatin, HYS = hysterectomy, IC = ifosfamide and cisplatin, LND = lymph node dissection, NA = not available, NACT = neoadjuvant chemotherapy, PACT = postoperative adjuvant chemotherapy, PC = paclitaxel and carboplatin, PFS = progression-free survival, RT = radiation therapy.

∗ CA125 refers to the level of serum CA125 before surgery.

† PFS2 refers to the time interval from second surgery to the first radiologic or biopsy-proven disease recurrence.

The median age of these patients when diagnosed with MNAC or MLA was 52 years old, ranging from 29 to 75 years old. Among them, the median age of patients with uterine MLA was 62 years old. As for clinical presentation observed in this study, patients with ovarian MLA or oviducal MNAC initially presented with abdominal pain or pelvic pain, patients with uterine MLA and cervical MNAC initially presented with vaginal bleeding, and patient with vaginal MNAC initially presented with vaginal discomfort. Nine of the 11 patients had a pre-operative CA-125 drawn with 1 patient having an elevated measurement (Case number 5, Table 1). Clinical stage among these patients was IA (1 patient), IB (5 patients), ifosfamide and cisplatin (2 patients), IIIA (1 patients), IIIC (1 patient), and IVB (1 patient).

**Figure 1:**
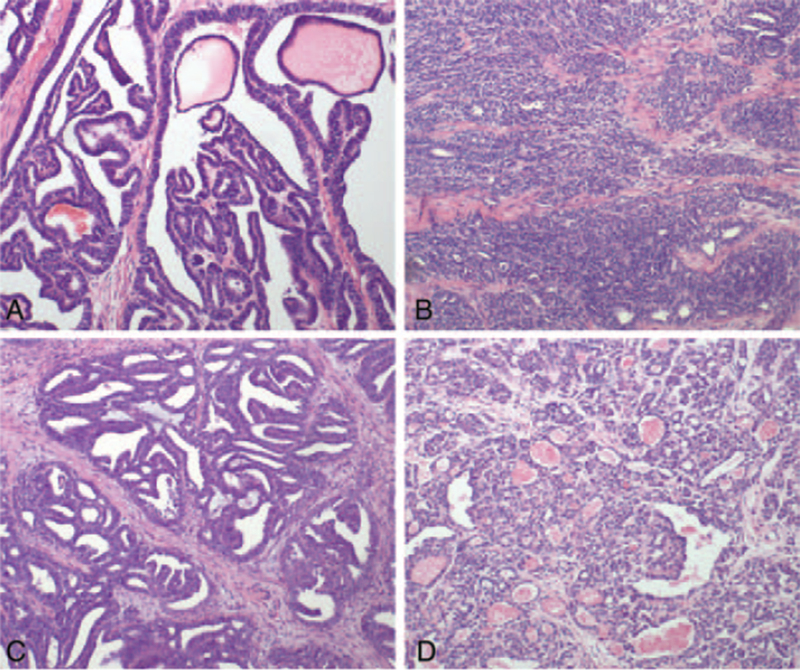
In most of the cases, there was a prominent glandular and tubular architecture, some of the tubules containing luminal eosinophilic colloid-like material (A). In some of the cases, mesonephric adenocarcinomas or mesonephric-like adenocarcinomas may show solid (B) and glandular (C). Some of the tumors exhibited mainly solid and a focal glandular architecture (D). Staining method: hematoxylin-eosin [HE] staining. Original magnification: (A) ×200, (B) ×40, (C) ×100, (D) ×100.

**Figure 2:**
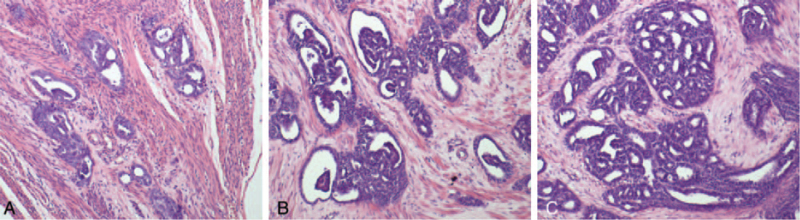
In the oviducal mesonephric adenocarcinoma, mesonephric remnants (A), mesonephric hyperplasia (B) and hyperplasia into cancerous nests (C) were histologically found. Staining method: hematoxylin-eosin [HE] staining. Original magnification: (A–C) ×100.

**Figure 3:**
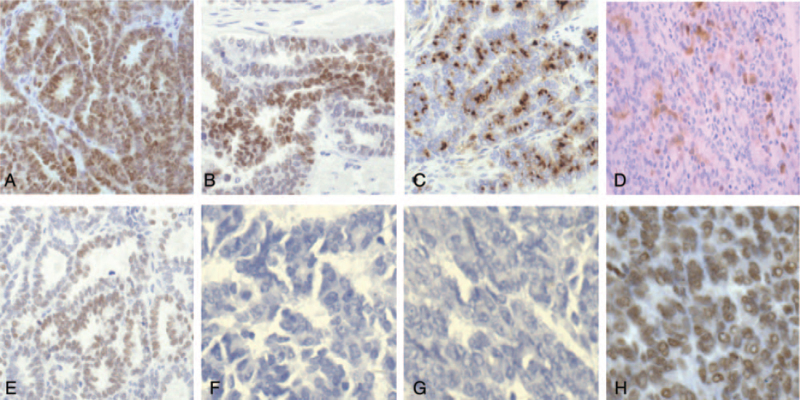
Immunohistochemically, all cases tested were positive with PAX8 (A). Ten of 11, 7 of 11, and 9 of 11 cases exhibited some degree of immunoreactivity with GATA3 (B), CD10 (C), and calretinin (D), respectively. In all CD10-positive cases, the staining was luminal. Ten of 11 cases were positive with TTF1 (E). All tumors were totally negative with ER (F) and PR (G), and all tumors exhibited wild-type staining with p53 (H). Staining method: immunohistochemical staining. Original magnification: (A–E) ×200, (F–H) ×400.

As for tumor histology, the pathologic features for MNAC or MLA are characterized histologically by a variety of architectural patterns such as glandular and tubular, glandular, and solid in various combinations. In most of the cases (6/11 cases), there was a prominent glandular and tubular architecture, some of the tubules containing luminal eosinophilic colloid-like material (Fig. 1A). In some of the cases (3/11 cases), MNACs or MLA may show solid (Fig. 1B) and glandular (Fig. 1C). Some of the tumors (2/11 cases) exhibited mainly solid and a focal glandular architecture (Fig. 1D). Mesonephric remnants (Fig. 2A), mesonephric hyperplasia (Fig. 2B) and hyperplasia into cancerous nests (Fig. 2C) were histologically found in the oviducal case, while no mesonephric remnants was found in any of the 2 ovarian cases. Immunohistochemically, all cases tested were positive with PAX8 (Fig. 3A). Ten of 11, 7 of 11, and 9 of 11 cases exhibited some degree of immunoreactivity with GATA3 (Fig. 3B), CD10 (Fig. 3C), and calretinin (Fig. 3D), respectively. In all CD10-positive cases, the staining was luminal. Most of the cases exhibited nuclear TTF-1 expression (10/11 cases were positive, Fig. 3E). All cases were totally negative with ER (Fig. 3F) and PR (Fig. 3G), and all tumors exhibited “wild type” immunoreactivity with p53 (Fig. 3H). All of the uterine neoplasms pre-dominantly involved the endometrium where they appeared to arise, with subsequent invasion into the myometrium. None of uterine MLA involved the myometrium without endometrial involvement.

### 3.2 Treatment of MNACs in female genital tract

All patients underwent total hysterectomy and bilateral salpingo-oophorectomy, and patients with cervical MNAC underwent radical hysterectomy according to the treatment principle of cervical cancer. Omentectomy was performed in patients with ovarian MLA or MNAC of fallopian tube, and lymph node dissections were performed in 7/11 (63.6%) patients. Most of patients (8/11) achieved an R0 resection, and none of the patients with lymph node dissections were found to have positive nodal disease. According to surgical staging, 8/11 (72.7%) patients had stage 1 disease, 2/11 (18.2%) patients had stage 3 disease, 1 (9.1%) patient had stage 4 disease. Eight patients (72.7%) received postoperative adjuvant chemotherapy (PACT), while 3 early-stage patients had surgery only. Two/eleven (18.2%) received neoadjuvant chemotherapy (NACT) before surgery with docetaxel/carboplatin or ifosfamide/cisplatin, and 8/11 (72.7%) received adjuvant chemotherapy after primary surgery (Table 1). NACT was performed in the patients with uterine MNAC, whose staging system of the International Federation of Gynecology and Obstetrics (FIGO stage) were IIIC and IVB. Chemotherapeutic agents included paclitaxel, cisplatin, carboplatin, ifosfamide, gemcitabine, and docetaxel. Regimens based on paclitaxel and platinum-derivatives formed the main body of the combination of NACT and PACT. Of the 8 patients who received PACT, 6/8 (75.0%) received 4 to 8 cycles of carboplatin/paclitaxel, 1/8 (12.5%) received 6 cycles of carboplatin/docetaxel, and 1/8 (12.5%) received 6 cycles of ifosfamide/cisplatin. Of the 11 patients, only 1 patient received adjuvant radiation therapy (Case number 5, Table 1). The patient was diagnosed with uterine MNAC and had pulmonary metastasis when initial diagnosis, she received adjuvant radiation therapy and chemotherapy after surgery. All cervical MNAC patients underwent radical hysterectomy and pelvic plus para-aortic lymphadenectomy, and systemic chemotherapy (neoadjuvant or postoperative chemotherapy) was not performed for both of the 2 patients (Case number 10 and 11, Table 1). Among the 5 uterine MNACs, 2 received adjuvant chemotherapy of paclitaxel and carboplatin, 1 with FIGO stage IIIA received adjuvant chemotherapy of docetaxel and carboplatin, 1 with FIGO stage IVB received adjuvant chemotherapy of ifosfamide and cisplatin, and 1 with FIGO stage I received no adjuvant treatment (Case number 4–8, Table 1).

### 3.3 Outcome of MNACs in the female genital tract

Up to now, 1 patient (9.1%) died at the end point of this study, and 10 patients (90.9%) survived, among whom 4 patients survived more than 5 years. All the 4 patients who survived more than 5 years had stage I disease, among whom 3 patients are still free of disease. In addition, 1 patient diagnosed with vaginal MNAC survived more than 8 years. Among the 11 patients, 3 patients have relapsed, including 2 patients with distant metastases and 1 patient with locoregional recurrence. Of the 2 patients who recurred with distant disease, 1 died before receiving salvage treatment (Case number 5, Table 1), 1 is still alive with no evidence of disease after the second resection of metastatic lesions followed by a new chemotherapy combination with gemcitabine plus carboplatin (Case number 1, Table 1). The patient who presented with distant relapse developed hepatic recurrence after complete staging surgery combined with systemic chemotherapy, and the second resection of metastatic lesions followed by a new chemotherapy combination with gemcitabine plus carboplatinut was performed, and there is no evidence of disease with 22-month follow-up after the second surgery. The patient who presented with locoregional recurrence (Case number 8, Table 1) was lost to follow-up after her relapse (current disease status unknown). The median PFS for all the patients was 13 months (mean: 33.5 months; range: 8–90 months), and the median PFS of 5 patients with uterine MNAC was 11 months (mean: 20.4 months; range: 8–61 months).

## 4 Discussion

MNAC or MLA is an extremely rare and highly malignant tumor in the female genital tract. Currently, little is known about the clinical characteristics, pathological diagnosis, prognosis, and optimal management strategy of MNAC and MLA in the female reproductive system. Due to the rareness of the disease, current study about MNAC or MLA is very little and pre-dominantly limited to case reports. In addition, these studies have demonstrated inconsistent findings. To add to existing literature, we performed a retrospective review of 11 cases of MNAC or MLA diagnosed and treated at a single institution between 2010 and 2020, and this study aimed to summarize the clinical characteristics, pathological diagnosis, treatment, and prognosis of MNAC or MLA in female genital tract.

Most of MNACs in female genital tract are incidental findings. Neither MNAC nor MLA has specific clinical manifestations. In our study, MNACs occurred in the cervix or uterine corpus mainly presented with vaginal bleeding or vaginal discharge, and MNAC of the fallopian tube or ovarian MLA mainly presented with abdominal pain or discomfort. The levels of serum tumor markers in patients with MNAC or MLA may be within normal range. Nine of 11 patients had a pre-operative CA125 drawn with 1patient having an elevated measurement in this study (Table 1). Therefore, the pre-operative diagnosis of MNAC or MLA is extremely difficult and definite diagnosis almost depends on postoperative histology and immunohistochemistry.

Due to the paucity of available data, there is no established guideline about standard therapy of MNAC or MLA. However, a radical surgery is considered to be the initial choice for localized disease. In the current study, all the patients underwent hysterectomy and bilateral salpingo-oophorectomy, and 6/11 patients received lymphadenectomy. Of the 5 patients without lymphadenectomy, 2 patients were diagnosed with late stage MNAC (≥FIGO stage III), lymphadenectomy is not beneficial to these patients and may increase the risk associated with the operation. Omentectomy was performed in patients with ovarian MLA or MNAC of fallopian tube.

MNACs might be very aggressive, even when early stage. A recent study including 31 cases of MNAC reported that 82% of the patients were at FIGO stage IB when initial diagnosis, but one-third of the patients with FIGO stage I disease developed recurrence even after radical surgery. Distant metastases and local recurrence were frequent findings in this study, and the median time to recurrence was 2.1 years.^[10]^ In another study, the recurrence rate among patients with FIGO stage I MNAC was 32%.^[11]^ Distant metastases at initial diagnosis was found in less than 5% of MNAC cases, but a malignant clinical course has been reported in about 40% of patients.^[12,13]^ Recently, a multi-institutional study of 99 MAs and MLAs demonstrated that the majority of mesonephric neoplasms presented at an advanced stage (II–IV) (60% MA of the cervix, 58% MLA of the endometrium, and 39% MLA of the ovary), and the majority (52% overall) developed recurrences, with a pre-dilection for pulmonary recurrence.^[14]^ In our study, 8/11 patients were at FIGO stage I when initially diagnosed, and all the 8 patients underwent radical surgery. Of the 8 patients, 2 recurred. Of the 2 early stage patients with recurrence, 1 was oviducal MNAC and recurred 4 months after radical surgery followed by systemic chemotherapy (Case number 1, Table 1), 1 was uterine MNAC and developed recurrence 61 months after complete solo surgery (Case number 8, Table 1).

The role of adjuvant chemotherapy in early stage MNAC or MLA is unclear. In the current study, 8 patients had stage I disease, 2 of these patients did not receive adjuvant treatment after radical surgery. Both of the 2 patients were cervical MNAC and had no evidence of disease at the end point of this study. Of these patients with stage I disease who received systemic chemotherapy after surgery, 1 developed recurrence of the disease. The vast majority of MNAC or MLA patients who underwent adjuvant chemotherapy received 4 to 8 cycles of paclitaxel and carboplatin as first-line treatment. In this study, 2/11 (18.2%) received NACT prior to surgery, with the chemotherapy regimen of docetaxel and carboplatin and ifosfamide and cisplatin, respectively, and 7/11 (63.6%) received adjuvant chemotherapy after primary surgery (Table 1). NACT was performed in the patients with uterine MNAC, whose FIGO stage were IIIC and IV. Regimens based on paclitaxel and platinum-derivatives formed the main body of the combination of NACT and PACT. There was very few patients reported to treat with radiation. Of the 11 patients, only 1 patients in this study received adjuvant radiotherapy after surgery in this study. Radiation was very limited in the treatment of the disease.

There is very little research about chemotherapy regimen in recurrent MNAC. A recent study showed a recurrent MNAC case had good response to the combination chemotherapy of carboplatin and paclitaxel.^[15]^ Of the 3 patients who recurred in this study, 1 patient died before receiving salvage treatment, 1 was lost to follow-up immediately following being diagnosed with tumor recurrence, and 1 received second surgery followed by a new chemotherapy regimen. The patient who had been given chemotherapy with carboplatin plus paclitaxel after initial surgery, rapidly developed a distant relapse, hence a new chemotherapy combination with gemcitabine and carboplatin was administered to treat this patient after the second resection of metastatic lesions, and the patient remains with no evidence of disease recurrence with 22 months of follow-up. Based on the lack of patients in the current study, the role of chemotherapy in recurrent MNAC or MLA remains unclear, but this represents an area that warrants future research.

## 5 Conclusion

In this contemporary review of 11 patients with MNAC or MLA of the female genital tract, radical surgery is considered to be the initial choice for localized disease. Given the high malignancy, the majority of MNAC or MLA patients who underwent adjuvant chemotherapy received 4 to 8 cycles of carboplatin plus paclitaxel as first-line treatment after primary surgery. MNAC or MLA might be very aggressive, even when early stage. Recurrence rate was lower than expected for early stage disease. Treatment for recurrent disease in these patients included gemcitabine, carboplatin, and paclitaxel. Radiation is very limited in the treatment of the disease.

## Author contributions

**Conceptualization:** Chuan Xie.

**Data curation:** Chuan Xie, Qiuhe Chen.

**Formal analysis:** Chuan Xie.

**Investigation:** Chuan Xie.

**Methodology:** Chuan Xie, Yangmei Shen.

**Software:** Chuan Xie, Qiuhe Chen.

**Supervision:** Qiuhe Chen.

**Validation:** Chuan Xie, Qiuhe Chen.

**Visualization:** Qiuhe Chen.

**Writing – original draft:** Chuan Xie.

**Writing – review & editing:** Chuan Xie, Yangmei Shen.
